# Predicted Growth of Two-Dimensional Topological Insulator Thin Films of III-V Compounds on Si(111) Substrate

**DOI:** 10.1038/srep15463

**Published:** 2015-11-05

**Authors:** Liang-Zi Yao, Christian P. Crisostomo, Chun-Chen Yeh, Shu-Ming Lai, Zhi-Quan Huang, Chia-Hsiu Hsu, Feng-Chuan Chuang, Hsin Lin, Arun Bansil

**Affiliations:** 1 Department of Physics, National Sun Yat-Sen University, Kaohsiung 804, Taiwan; 2 Centre for Advanced 2D Materials and Graphene Research Centre, National University of Singapore, Singapore 117546; 3 Department of Physics, National University of Singapore, Singapore 117542; 4 Department of Physics, Northeastern University, Boston, Massachusetts 02115, USA

## Abstract

We have carried out systematic first-principles electronic structure computations of growth of ultrathin films of compounds of group III (B, Al, In, Ga, and Tl) with group V (N, P, As, Sb, and Bi) elements on Si(111) substrate, including effects of hydrogenation. Two bilayers (BLs) of AlBi, InBi, GaBi, TlAs, and TlSb are found to support a topological phase over a wide range of strains, in addition to BBi, TlN, and TlBi which can be driven into the nontrivial phase via strain. A large band gap of 134 meV is identified in hydrogenated 2 BL film of InBi. One and two BL films of GaBi and 2 BL films of InBi and TlAs on Si(111) surface possess nontrivial phases with a band gap as large as 121 meV in the case of 2 BL film of GaBi. Persistence of the nontrivial phase upon hydrogenations in the III-V thin films suggests that these films are suitable for growing on various substrates.

Topological insulators (TIs) in general, and the two-dimensional (2D) quantum spin Hall insulators in particular, have been receiving increasing attention in the last few years in both theoretical and experimental studies^1^,^2^. These materials support conducting edge states in 2D and conducting surface states in 3D, even though their bulk states are insulating. These unique properties make topological insulator materials well suited for spintronics, quantum computing, and other applications due to the robustness of their edge/surface states against backscattering. In fact, a variety of atomically thin films in the buckled honeycomb structure of groups IV (Si, Ge, Sn)^3^,^4^,^5^, V (Bi, Sb, As)^6^,^7^,^8^,^9^,^10^,^11^, and III-V^12^,^13^,^14^,^15^ elements have been theoretically predicted to harbor topological insulating phases.

In order to be realized experimentally, however, a film must eventually be placed or grown on a suitable substrate. Effects of substrates, or environments more generally, can be simulated via hydrogenation or halogenation, and/or strains. Numerous studies attempting to explore such effects^16^ include work on films of silicene^17^,^18^, stanene^19^,^20^, Bi, Sb^21^,^22^,^23^, and group III-V compounds^12^,^13^,^14^,^15^. These studies demonstrate that substrates can be expected to induce substantial modifications in the electronic structures and band topologies, adding to the challenge of synthesizing films which support nontrivial topological states, especially films with band gaps large enough for room temperature applications.

Although the layer or thickness dependency of topological properties of thin films of group IV^20^ and V^8^,^10^,^24^ elements has been reported, we are not aware of a previous study delineating how nontrivial phases evolve in thin films of III-V compounds as the number of layers increases; all the important effects of substrate on the III-V films^25^,^26^,^27^ have also not been investigated. With this motivation, here we explore the crystal and electronic structures of hydrogenated III-V films using first-principles calculations. Nontrivial phases are predicted in 2 BL thick films of hydrogenated AlBi, GaBi, InBi, TlAs, and TlSb, which are found to survive over a wide range of strains. BBi, TlN, and TlBi films support a nontrivial phase for certain values of strain. Nontrivial topological phases are also found in 1 and 2 BL films of GaBi as well as 2 BL films of InBi and TlAs on Si(111) substrate. For the 2 BL film of GaBi on Si(111), the band gap can be as large as 121 meV, well above the room-temperature thermal excitation energy. Si(111) would thus provide a viable substrate for growing the proposed topological III-V films.

## Results

**Figure 1:**
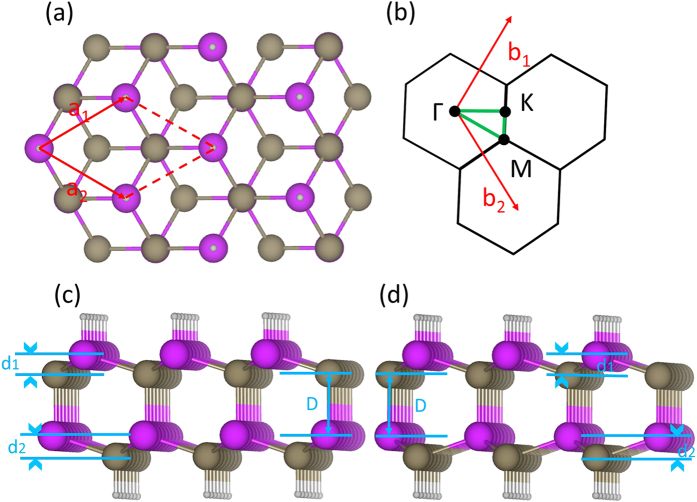
(**a**) Top view of the 2 BL hydrogenated zincblende structure; the 1 × 1 unit cell is outlined. (**b**) 2D Brillouin-zones with specific symmetry points labeled. (**c**–**d**) Side views of 2 BL films in ZB and WZ structures are shown in (**c**,**d**), respectively; the layer distance (*D*), and the buckling heights *d*_1_ and *d*_2_ in the top and bottom layer are marked.

We assumed the investigated III-V films to be stacked along the [111] direction in zincblende (ZB) and wurtzite (WZ) structures. We define one BL as a III-V pair of layers in the buckled honeycomb structure. The 2 BL film can assume ZB and WZ as two possible structures. The top view of the atomic structure (1 × 1 unit cell is outlined) for a 2 BL film based on the ZB structure is shown in Fig. 1(a). The surface Brillouin zone is presented in Fig. 1(b). Side views of a 2 BL film in ZB and WZ are shown in Fig. 1(c,d), respectively. The band structures for the equilibrium crystal structures were calculated including the spin-orbit coupling. In order to ascertain the band topologies, we computed the Z_2_ invariant following the method of ref. 28.

Previous studies^12^,^13^,^14^ have shown that the only 1 BL films of III-V compounds, which exhibit nontrivial phases, involve Bi from group V and Tl from group III. With this in mind, even though we investigated all 25 combinations of group III (B, Al, In, Ga, and Tl) and group V (N, P, As, Sb, and Bi) elements, we will focus on 2 BL films of Bi and Tl, many of which are 2D TIs in the freestanding case. The optimized structures of these nine 2 BL III-V films before and after hydrogenation are summarized in Table 1. Also included in Table 1 are the preferred structure, the system band gap (defined as the energy difference between the lowest conduction band and the highest valence band), and the *Z*_2_ invariant, along with the bulk lattice constant, *a*_0_, the optimized lattice constant, *a*, of the film, buckling distances in the upper (*d*_1_) and lower (*d*_2_) layer, and the distance (*D*) between the two layers in the 2 BL film. The hydrogenated 2 BL film of InBi is seen in Table 1 to possess the largest band gap of 134 meV at its equilibrium structure.

A reference to Table 1 shows that the unhydrogenated 2 BL films of AlBi, GaBi, and InBi are nontrivial semi-metals with *Z*_2_ = 1 and a negative system gap. TlN, TlAs, and TlSb, in contrast, are nontrivial insulators with band gaps of 18 to 51 meV. Upon hydrogenation, only the TlN film changes from nontrivial to trivial phase, where this transition is partially driven by the 4% (3.739 Å to 3.9 Å) change in lattice constant. Since all of the 2 BL films in Table 1 are seen to prefer the ZB rather than the WZ structure around the equilibrium bulk lattice constant, for the remainder of this study, we will only consider the ZB structure.

**Figure 2:**
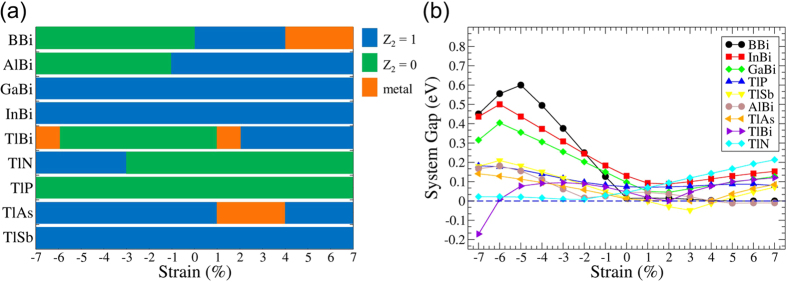
(**a**) Evolution of the topological phase as a function of strain in selected hydrogenated 2 BL films, and (**b**) the corresponding system band gaps as a function of strain.

In order to simulate the effects of the supporting substrate, nine hydrogenated films with ZB structure in Table 1 were further analyzed over a range of lateral strains around the equilibrium structures (0% strain); the results are summarized in Fig. 2. We see from Fig. 2(a) that the hydrogenated 2 BL films of TlAs, TlSb, AlBi, GaBi, and InBi maintain their nontrivial phase over a wide range of strains; in fact, certain strain values yield even larger band gaps, see Fig. 2(b). The TlBi film, even though it is trivial phase at its equilibrium lattice constant, exhibits nontrivial phase at strain values greater than 2% and less than −8%. It is remarkable that the nontrivial phase is robust for selected hydrogenated III-V films in that it is maintained over a wide range of strains, suggesting that this nontrivial phase is more likely to be realized when the film is grown on a suitable substrate. Notably, this family of films harbors band gaps large enough for possible room temperature applications. InBi and GaBi films, for example, possess band gaps up to 0.4 eV under compressive strain of around −5%.

**Figure 3:**
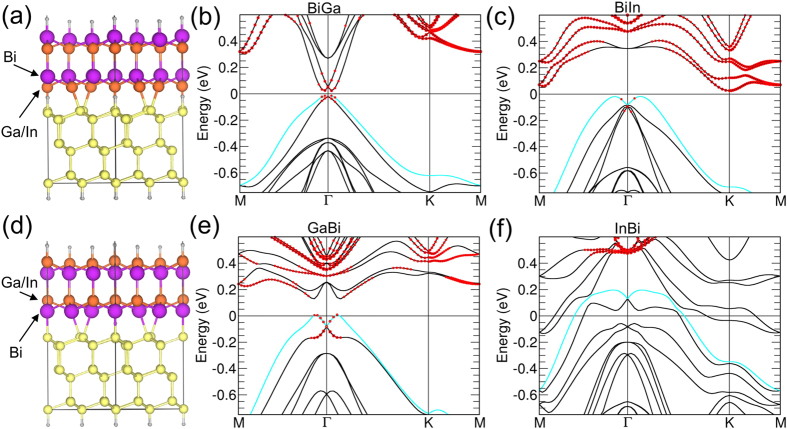
(**a**,**d**) Crystal structure of 2 BL films of Ga/In-Bi on Si(111). Bi layer is on the top in (**a**) with Ga/In below, while in (**d**), Ga/In layer lies on top of the Bi layer. (**b**,**c**) are band structures of GaBi and InBi, respectively, provided that In or Ga bonds with Si(111), whereas (**e**,**f**) are respectively for Bi bonding with Si. The size of red circles is proportional to the contribution of the *s*-orbital. The cyan line indicates the highest valence band.

Turning to the question of practical realization of our proposed films, Si(111), may be a good candidate substrate to grow TlAs, AlBi, GaBi, and InBi films since the hydrogenated equilibrium lattice constants of these 2 BL films in 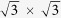 supercell (7.85, 7.95, 7.93, and 8.44 Å) are close to the lattice constant (7.73 Å) of 2 × 2 Si(111); also, as noted already in connection with Fig. 2 above, GaBi and InBi films maintain their nontrivial topology over a wide range of strains, while TlAs and AlBi do so near their equilibrium lattice constants. Note that the 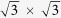 surfaces of InBi and GaBi have lattice mismatches of −8.41% and −2.52% with respect to the 2 × 2 Si(111) surface, obtained by using 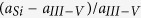, where *a*_*Si*_ and *a*_*III−V*_ are the equilibrium lattice constants of 2 × 2 Si(111) and hydrogenated 2 BL III-V film, respectively. In this connection, we further explored substrate effects by placing InBi and GaBi films on the Si(111) substrate. For this purpose, three Si(111) bilayers were used to simulate the substrate with the bottom Si(111) layer passivated with H atom. The Si atoms at the bottom bilayer and the passivating H atoms were fixed while all other atoms were allowed to relax. [In these calculations, a Γ-centered Monkhorst-Pack^29^ grid of 12 × 12 × 1 in the 2D Brillouin zone of 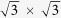 buckled honeycomb was used.] Since III-V films contain two distinct surfaces, two different types of bonding with the substrate is possible. The Si(111) layer can bond either to the Bi layer or In/Ga atoms. Figure 3 presents the crystal and band structures for these two cases in which the Bi layer lies on (a) top or (d) below the Ga/In layer. The corresponding band structures for Bi on top of Ga (b) and In (c) and for Bi below Ga (e) and In (f) are also shown. Table 2 summarizes the main results by giving the total energy, the system energy gap, and the *Z*_2_ invariant for 1 BL and 2 BL Ga/In-Bi, AlBi, and TlAs films on Si(111). For 2 BL Ga/In-Bi films, both cases support nontrivial topological insulator phase and a band gap [121 meV for GaBi-Si(111)] large enough for room-temperature applications. Notably, 1 BL films of GaBi or BiGa on Si(111) also exhibit a nontrivial topological phase. The transition to trivial from nontrivial phase of 1 BL film of InBi or BiIn on Si(111) is consistent with our previous work^13^ in which the hydrogenated 1 BL film of InBi was found to be trivial under –4% strain. Finally, we comment on the results for AlBi and TlAs films. As shown in Fig. 2(a), 2 BL film of AlBi becomes trivial below −1% strain, while TlAs film maintains the nontrivial phase for less than 1% strain. [AlBi and TlAs have lattice mismatches of −2.77% and −1.53%, respectively.] When placed on Si(111), only the TlAs film exhibits a nontrivial phase as expected in view of its lattice mismatch with Si(111), see also Fig. 2. We note that all 1 BL films of AlBi and TlAs films are found to be trivial.

## Conclusions

We have presented a first-principles study of the crystal and electronic structures of freestanding and hydrogenated multilayers of III-V compounds. Two BL films of hydrogenated AlBi, GaBi, InBi, TlAs, and TlSb are found to harbor the nontrivial insulator phase. BBi, TlN, and TlBi films can be driven into the nontrivial topological phase via strain, although these films are in the trivial phase at their equilibrium structures. Hydrogenated 2 BL films of GaBi and InBi exhibit band gaps of 99 meV and 134 meV, respectively. We have also explored the electronic structures and topological properties of 1 BL and 2 BL films of GaBi as well as 2 BL films of InBi and TlAs on a Si(111) substrate, and found a nontrivial phase with a large band gap of 121 meV in 2 BL films of GaBi. Our study suggests that III-V thin films can support nontrivial topological phases, which are robust against hydrogenation, strain and substrate effects, and would thus provide a viable materials platform for future room temperature applications.

## Methods

First-principles calculations were performed within the density functional theory (DFT) utilizing the generalized gradient approximation (GGA)^30^,^31^,^32^,^33^,^34^. Projector-augmented-wave (PAW)^35^ wave functions with an energy cut-off of 400 eV were used in the Vienna Ab-Initio Simulation Package (VASP)^36^,^37^. Atomic positions were optimized for each lattice constant value considered until the residual forces were no greater than 10^−3^ eV/Å. The criteria for convergence for self-consistent electronic structure was set at 10^−6^ eV. A vacuum layer of at least 20 Å along the *z* direction was used to simulate multilayer films. A Γ-centered Monkhorst-Pack^29^ grid of 18 × 18 × 1 was used for 2D integrations in the Brillouin zone of the 1 × 1 buckled honeycomb lattice.

## Additional Information

**How to cite this article**: Chuang, F.-C. *et al.* Predicted Growth of Two-Dimensional Topological Insulator Thin Films of III-V Compounds on Si(111) Substrate. *Sci. Rep.* **5**, 15463; doi: 10.1038/srep15463 (2015).

## Figures and Tables

**Table 1:** Calculated equilibrium structure [zincblende (ZB)], system band gap defined as the energy difference between the lowest conduction level and the highest valence level, and the topological invariant *Z*_2_ for nine 2 BL films of III-V compounds with and without hydrogenation.

	Material	Structure	Sys. Gap (meV)	*Z*_2_	*a*_0_(Å)	*a* (Å)	*d*_1_(Å)	*d*_2_ (Å)	*D* (Å)
w/o H	BBi	ZB	−366	0	3.90	3.90	0.56	0.80	2.58
	AlBi	ZB	−118	1	4.83	4.56	0.78	0.85	2.98
	GaBi	ZB	−67	1	4.56	4.55	0.78	0.86	2.98
	InBi	ZB	−43	1	4.84	4.85	0.82	0.91	3.15
	TlN	ZB	18	1	3.78	3.95	0.14	0.15	2.37
	TlP	ZB	−41	0	4.39	4.42	0.68	0.74	2.97
	TlAs	ZB	51	1	4.53	4.56	0.73	0.82	3.02
	TlSb	ZB	42	1	4.83	4.85	0.80	0.95	3.15
	TlBi	ZB	−59	0	4.96	4.98	0.75	0.96	3.19
w/H	BBi	ZB	13	0	3.90	3.93	0.70	0.73	2.40
	AlBi	ZB	44	1	4.83	4.59	0.91	0.87	2.74
	GaBi	ZB	99	1	4.56	4.58	0.91	0.86	2.73
	InBi	ZB	134	1	4.84	4.87	0.99	0.91	2.90
	TlN	ZB	41	0	3.81	3.74	0.92	0.84	2.25
	TlP	ZB	78	0	4.39	4.38	0.96	0.90	2.61
	TlAs	ZB	24	1	4.53	4.53	0.98	0.91	2.70
	TlSb	ZB	12	1	4.83	4.81	1.00	0.96	2.88
	TlBi	ZB	47	0	4.96	4.97	1.01	0.94	2.96

Other structural parameters given are: bulk lattice constant (*a*_0_); lattice constant *a* of the 2 BL film; interlayer distance *D* in the 2 BL film; and the buckling heights *d*_1_ and *d*_2_ of the two layers. Distances *D, d*_1_ and *d*_2_ are also marked in Fig. 1.

**Table 2:** Total energy, system gap, and the topological invariant (*Z*_2_) of 1 and 2 BL films of Ga/In-Bi, AlBi, and TlAs on Si(111).

Material		Total energy(eV)	System Gap(meV)	Z_2_
2BL	BiGa-Si(111)	−196.514	37	1
	GaBi-Si(111)	−197.146	121	1
	BiIn-Si(111)	−193.410	44	1
	InBi-Si(111)	−194.443	−330	1
	AlBi-Si(111)	−201.291	−16	*m*
	BiAl-Si(111)	−200.415	178	0
	TlAs-Si(111)	−192.820	76	1
	AsTl-Si(111)	−194.200	158	0
1BL	BiGa-Si(111)	−174.793	68	1
	GaBi-Si(111)	−175.324	84	1
	BiIn-Si(111)	−173.291	445	0
	InBi-Si(111)	−173.327	254	0
	AlBi-Si(111)	−177.584	466	0
	BiAl-Si(111)	−176.842	611	0
	TlAs-Si(111)	−173.012	195	0
	AsTl-Si(111)	−174.198	342	0

*Z*_2_ is 1 for nontrivial and 0 for trivial band topology, while *m* denotes that the system is metallic.
